# Temporal Audiovisual Motion Prediction in 2D- vs. 3D-Environments

**DOI:** 10.3389/fpsyg.2018.00368

**Published:** 2018-03-21

**Authors:** Sandra Dittrich, Tömme Noesselt

**Affiliations:** ^1^Department of Biological Psychology, Otto von Guericke University Magdeburg, Magdeburg, Germany; ^2^Center for Behavioral Brain Sciences, Magdeburg, Germany

**Keywords:** audiovisual, multisensory integration, motion prediction, motion in depth, individual differences

## Abstract

Predicting motion is essential for many everyday life activities, e.g., in road traffic. Previous studies on motion prediction failed to find consistent results, which might be due to the use of very different stimulus material and behavioural tasks. Here, we directly tested the influence of task (detection, extrapolation) and stimulus features (visual vs. audiovisual and three-dimensional vs. non-three-dimensional) on temporal motion prediction in two psychophysical experiments. In both experiments a ball followed a trajectory toward the observer and temporarily disappeared behind an occluder. In audiovisual conditions a moving white noise (congruent or non-congruent to visual motion direction) was presented concurrently. In experiment 1 the ball reappeared on a predictable or a non-predictable trajectory and participants detected when the ball reappeared. In experiment 2 the ball did not reappear after occlusion and participants judged when the ball would reach a specified position at two possible distances from the occluder (extrapolation task). Both experiments were conducted in three-dimensional space (using stereoscopic screen and polarised glasses) and also without stereoscopic presentation. Participants benefitted from visually predictable trajectories and concurrent sounds during detection. Additionally, visual facilitation was more pronounced for non-3D stimulation during detection task. In contrast, for a more complex extrapolation task group mean results indicated that auditory information impaired motion prediction. However, a *post hoc* cross-validation procedure (split-half) revealed that participants varied in their ability to use sounds during motion extrapolation. Most participants selectively profited from either near or far extrapolation distances but were impaired for the other one. We propose that interindividual differences in extrapolation efficiency might be the mechanism governing this effect. Together, our results indicate that both a realistic experimental environment and subject-specific differences modulate the ability of audiovisual motion prediction and need to be considered in future research.

## Introduction

Motion prediction is a critical ability for many species, e.g., when catching prey or avoiding being caught by a predator. Even in humans this ability still governs behaviour in everyday traffic and sport, e.g., when driving a car, crossing a road or catching a ball. Previous studies on motion prediction predominantly focused on different tasks (e.g., time-to-arrival, Schiff and Oldak, 1990; same-different-discrimination, Kawachi and Gyoba, 2006; predicted motion, Prime and Harris, 2010) and the visual modality (e.g., DeLucia, 2004; Lugtigheid and Welchman, 2011; Landwehr et al., 2013).

However, many real-life events stimulate more than one single sensory modality. Accordingly, research recently started to focus on multisensory interactions in motion perception (though often with simple stimuli, Hofbauer et al., 2004; Prime and Harris, 2010). These studies reported that participants react faster when motion is presented in both modalities (Harrison et al., 2010) and showed enhanced perceptual sensitivity for bimodal than unimodal motion signals (Wuerger et al., 2003). Moreover, a salient motion signal in one modality can bias perception of a stationary or ambiguously moving stimuli in another modality (Hidaka et al., 2009; Teramoto et al., 2010; Alink et al., 2012).

Another line of research focuses on multisensory interplay in situations when a moving object is temporarily occluded, i.e., not always visible, and participants predict the object’s movement after occlusion as well. Already infants appear to benefit from additional dynamic auditory information during occlusion (Bremner et al., 2012) and their oculomotor anticipations are more pronounced for audiovisual than visual motion information while the object is occluded (Kirkham et al., 2012). However, systematic research on audiovisual motion prediction in human adults is scarce and inconsistent despite its ecological validity and critical role in everyday life situations.

Whereas audiovisual information facilitates performance when localising a moving object (Hofbauer et al., 2004), participants are in contrast not better in extrapolating the time of an object’s arrival at a certain position if they are provided with audiovisual motion cues compared to visual cues alone (Hofbauer et al., 2004). Another study reported that performance is enhanced for audiovisual relative to unisensory stimulation if a prediction of the end point of a trajectory is required (Prime and Harris, 2010). In contrast, another study which used realistic film clips of moving vehicles found no evidence that participants could benefit from additional auditory information compared to visual-only clips (Schiff and Oldak, 1990).

Differences in stimulus material and in particular the use of non-realistic simplified stimuli may be one reason for these variability in results. Most audiovisual motion studies used flashes (Hofbauer et al., 2004; Freeman and Driver, 2008; Hidaka et al., 2009; Harrison et al., 2010; Wuerger et al., 2010), gratings (Maeda et al., 2004; Jain et al., 2008), random dot kinematograms (Meyer and Wuerger, 2001; Alais and Burr, 2004; Baumann and Greenlee, 2007; Gleiss and Kayser, 2014) or simple geometric stimuli (Freeman and Driver, 2008; Hidaka et al., 2009; Prime and Harris, 2010; Bremner et al., 2012; Kirkham et al., 2012; Chien et al., 2013) combined with beeps (Freeman and Driver, 2008), clicks (Hofbauer et al., 2004; Mays and Schirillo, 2005; Wuerger et al., 2010), white noise bursts (Hidaka et al., 2009; Harrison et al., 2010; Teramoto et al., 2010) or intensity modulated pure tones from two speakers (Prime and Harris, 2010) for auditory co-stimulation. Other studies which have used more realistic video material of moving vehicles (Schiff and Oldak, 1990; Gordon and Rosenblum, 2005) indeed lacked stimulus control.

This absence of well-controlled studies with ecologically valid stimulation is especially problematic as a number of recent studies point to distinct behavioural and brain responses to realistic stimuli compared to their simplified alternatives: For instance, it has been reported that naturalistic auditory stimuli lead to faster reactions times (RTs) and earlier event-related potential (ERP) responses (Getzmann and Lewald, 2010). Concordantly, a recent functional magnetic resonance imaging (fMRI) study observed distinct response patterns for real objects compared to two-dimensional (2D) images of the same objects (Snow et al., 2011). For multisensory stimulation visual looming bias is intensified by looming sounds and this effect is further enhanced for more naturalistic Shepard-Stimuli (Conrad et al., 2013). Similarly, ERP-responses to naturalistic multisensory stimuli have a lower latency compared to abstract stimuli (Senkowski et al., 2007).

Due to the relevance of binocular cues to motion perception in depth (Cumming and Parker, 1994; Brooks and Stone, 2004; Rokers et al., 2009) three-dimensional (3D) stimulation, induced by visual disparity could be another critical ecological factor governing these differences (Kitagawa and Ichihara, 2002; Zannoli et al., 2012; Ogawa and Macaluso, 2013; Ogawa et al., 2013; Gaebler et al., 2014; Harrison et al., 2015). In fMRI studies distinct activation patterns were found while watching audiovisual movements (Ogawa and Macaluso, 2013) or movies (Ogawa et al., 2013) in 3D compared to 2D condition. Participants also report that a 3D version of the same movie is perceived as more immersive which is also accompanied by higher intersubject correlations of cortical networks in multivariate analysis (Gaebler et al., 2014).

Moreover, audiovisual interactions in depth have also been investigated with approaching (looming) and receding stimuli. Adaption to a visual looming stimulus led to a motion after effect for a stationary sound (Kitagawa and Ichihara, 2002), participants responded faster to bimodal than unimodal looming stimuli (Cappe et al., 2009) and there is also an advantage in visual search in depth if a search is accompanied by a congruent sound (Zannoli et al., 2012). Furthermore, the congruency effect (higher accuracies for audiovisual congruent looming conditions) has been reported to be more pronounced during stereoscopic 3D than 2D stimulation (Harrison et al., 2015).

In this study we tested the interplay between motion processing and different task demands (stimulus detection and motion extrapolation) in visual vs. audiovisual contexts. In the experiments described here, a stereoscopic 3D scenario was used by modifying the ball-in-a-box-paradigm (Kersten et al., 1997): A ball moved from the top of a box toward the participants. During movement the ball was temporarily occluded by a bridge and could reappear on a visually congruent or incongruent trajectory (experiment 1). Concurrent auditory motion could occur either in the same or a different direction as the ball (plus a visual-only condition without auditory co-stimulation).

In experiment 1 participants performed a simple detection task and indicated via button press when the ball reappeared after occlusion (temporal detection task). In experiment 2 we slightly modified our visual scene to perform a higher demanding extrapolation task. The ball did not reappear after occlusion and participants indicated when the ball would reach a red bar on a near or far distance after occlusion (temporal extrapolation task). We hypothesised, that predicting visual motion should be enhanced by additional auditory information and that this might further interact with stereoscopic vs. non-stereoscopic 3D stimulation.

## Materials and Methods

### Participants

All participants had normal vision (i.e., they reported no myopia, hyperopia, colour vision deficiency, or strabismus). None of the participants was stereo blind (Lang-Stereotest II, Lang-Stereotest AG Switzerland) and all reported to be without hearing deficits and history of neurological or psychiatric diseases. For participation, they received a fixed amount of money or were compensated with course credit. Participants gave their written informed consent and all experiments were conducted in accordance with the local ethical committee. For all experiments sex and experimental order of pseudo-3D and real-3D sessions were counterbalanced.

Forty-five volunteers participated in experiment 1. Nine participants had to be excluded. Eight responded too quickly in at least one of the experimental sessions (mean RT < 120 ms), another failed to follow the experimenter’s instructions. All excluded participants were replaced directly after the experiment to keep intact the counterbalancing. Data from 36 participants (18 female/male; mean age 22.50 ± 2.49 years) were analysed. Two participants were left-, one mixed- and 33 right-handed (Oldfield, 1971). In the second experiment 32 volunteers participated (16 female/male; mean age 23.34 ± 3.70 years). Three participants were left-, one mixed- and 28 right-handed (Oldfield, 1971).

### Stimulus Material and Procedures

Stimuli were presented using Matlab (R2012b, 8.00.783; The MathWorks, Inc., United States) and Psychophysics Toolbox 3.011 (Brainard, 1997; Kleiner et al., 2007) and were displayed on a Planar SD2220W Stereoscopic Monitor (Planar Systems, Inc., United States). The 3D impression was generated by using two vertically mounted monitors separated by a passive beam-splitter mirror while participants wore polarised glasses. Both screens (21.6″) had a resolution of 1920 × 1080 and a refresh rate of 60 Hz. Participants were placed 50 cm in front of the lower screen with their head resting on a chin rest. During real-3D session images had an offset (between 0.69° and 2.98°, determined in a pre-test) leading to an immediate 3D impression. Responses were collected via an USB-Keyboard (Damian, 2010).

**FIGURE 1:**
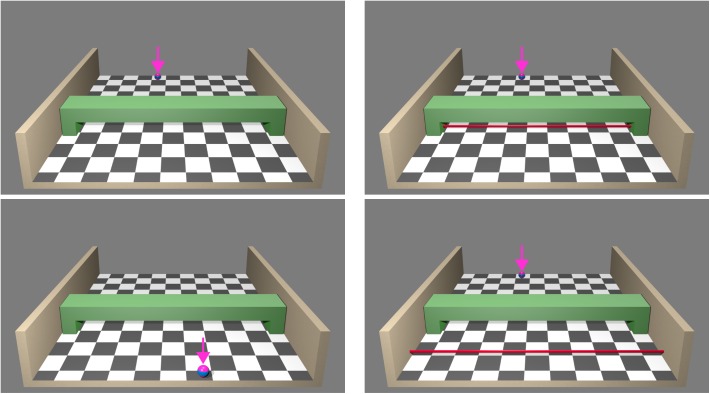
Stimulus material used in experiments 1 (left) and 2 (right). A ball (highlighted with pink arrows for illustrative purposes but not shown during the experiment) moved from the top of a box toward the participant. During movement the ball was temporarily occluded by the green bridge and could reappear following the identical or a divergent trajectory (experiment 1). Visual stimuli could be displayed in pseudo-3D and real-3D (varied session-wise and counterbalanced across participants). In both sessions stimuli were presented on a stereoscopic screen. In the real-3D sessions images had a spatial offset so that a 3D impression occurred when participants wore polarised glasses. There was no offset in pseudo-3D sessions. Auditory co-stimulation was a moving white noise either with the same or opposite direction as the visual trajectory. In addition, a visual-only condition was introduced. Participants detected when the ball reappeared (experiment 1, temporal detection). In experiment 2 the ball did not reappear after occlusion. Participants’ task was to indicate when the ball would have reached the red bar at a near or far position (temporal extrapolation).

**FIGURE 2:**
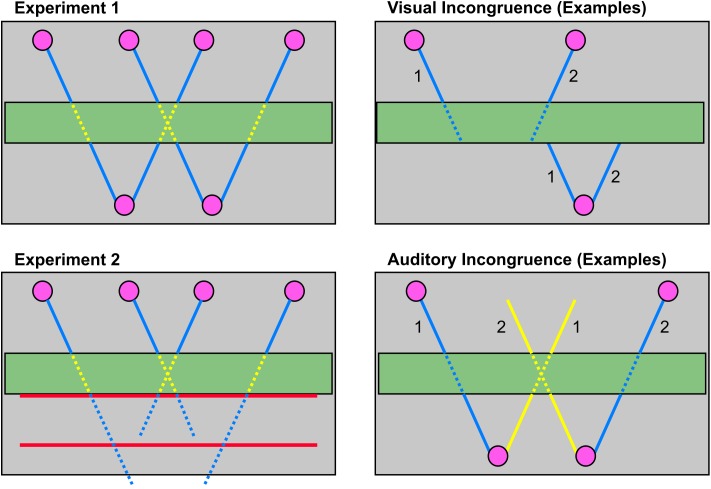
Illustration of visual trajectories and auditory stimulation (left) as well as visual and auditory incongruent trials (right). The left panel shows all 4 visual trajectories (left, middle left, middle right, right) viewed from above. Continuous lines illustrate a visible moving ball whereas dotted lines represent invisible ball movements (during occlusion in experiment 1, for experiment 2 as well after occlusion). In audiovisual trials the ball’s movement was accompanied by sounds (blue). During occlusion period the sound was attenuated (yellow). During audiovisual trials sounds always started with the beginning of visual motion. Auditory motion ended with the ball’s movement in experiment 1. In experiment 2 the auditory movement continued for 1533 ms after the ball would have reached the red bar for near and far extrapolation (illustrated by different line lengths for the outer and inner trajectories in the lower left panel). During visual incongruent trials the ball did not reappear on a consistent path after occlusion but rather reappeared on the second possible path for that direction. See upper right panel for two examples: Ball 1 started on the left, ball 2 on the middle right trajectory. In visual incongruent trials ball 1 reappeared on the middle left and ball 2 on the right trajectory. For auditory incongruent trials a sound was presented that moved in the opposite direction as the visual stimulation (lower right panel). Two examples are illustrated here: Ball 1 moves on the visual left, ball 2 on the right trajectory, ball 1 is accompanied by a sound moving on the middle right trajectory, ball 2 is accompanied by a sound on the middle left trajectory. To keep constant the distance of inconsistent auditory motion an outer visual trajectory was always paired with a middle trajectory moving in the opposite direction as the visual movement and the middle trajectories were paired with inconsistent outer auditory trajectories, respectively.

Visual stimulus material was created with Blender 2.75a^1^. The virtual scene consisted of a half-open box (width: 23.94°–45.08°, depth: 16.50°–20.96°) with a checkerboard patterned floor in front of a uniform grey background (**Figure 1** left). In the middle of the box a green bridge served as an occluding element (width: 31.82°, occluding depth part: 4.24°). All images contained pseudo-3D information (central perspective depth cues and shadows from a frontal light source). A deep-pink ball with a thick horizontal blue stripe (sized 1.15° at start of movement) moved from the rear toward the observer (sized 1.83° at end of movement). The ball could move along four different trajectories. Starting positions were located at ± 6.41° and ± 1.83°, ending positions at ± 2.86° left/right to box centre (note that ending positions for left and middle right/right and middle left trajectories were similar, all trajectories are illustrated in **Figure 2**). In the second experiment (extrapolation) the ball did not reappear after occlusion. For extrapolation a thin red bar (0.34° width) served as response cue, either at a distance of 0.46° or 4.92° from the bridge (**Figure 1** right) toward the observer.

During audiovisual stimulation the ball’s movement was accompanied by a continuous moving white noise (frequency range: 1000–2500 Hz). Auditory spatial information was delivered via four speakers mounted on the four corners of an imaginary rectangle between the lower monitor and the participant’s head to create an auditory 3D impression. Speakers were 60 cm separated horizontally, 35 cm in depth and mounted at head height (see Supplementary Figure S1 for a depiction of the speaker set-up). Diagonal auditory motion toward the observer was modelled with the help of different sound levels [mean sound pressure level 78 dB(A)]. Sound was attenuated during the occlusion period by 5 dB(A) (**Figure 2**). During the extrapolation task the sound stopped at the same time as visual stimulation, i.e., 1533 ms after the now invisible ball would have reached the thin red bar which served as a response cue. Thus, the end of the sound did not provide any additional temporal information in either of the tasks. Also note that results from an auditory-only pilot experiment indicated that participants (*n* = 10) were able to discriminate the auditory motion direction in the majority of trials (99.2% accuracy). Moreover, 9 out of 10 participants were able to judge whether the sound followed the central or peripheral trajectory (87.5% accuracy).

Pseudo-3D and real-3D stimuli were presented consecutively in two sessions. During both sessions participants were stimulated with images via both monitors and wore polarised glasses. However, only in the real-3D session images had an offset so that a true 3D impression could occur. During a 3D adjustment procedure prior to the real-3D session participants chose an offset for the 3D part out of 6 different offsets between upper and lower image to assure a maximal 3D impression per subject. All sessions started with 6 practise trials followed by 16 experimental blocks with 24 trials each. In experiment 1 the ball could reappear on a congruent or incongruent path after occlusion (**Figure 2**). Furthermore, there were conditions with no sound, with a sound movement congruent to the direction of the ball’s movement or with an incongruent sound movement direction (**Figure 2**). In experiment 2 the ball did not reappear after occlusion and participants had to extrapolate the ball’s movement at either a near or a far distance. Prior to extrapolation task in experiment 2 participants watched a demo with full, i.e., non-occluded, movements of all trajectories.

Each trial started with the ball resting at its start position for 200 ms. Afterwards it moved for 4167 ms toward the observer in experiment 1. During movement the ball was occluded for 567 ms in experiment 1. In experiment 2 movement was only visible before occlusion and the whole scene kept visible for 4000 ms (near extrapolation) or 4667 ms (far extrapolation) after the ball started to move, so that response time interval was identical for both extrapolation conditions at 1533 ms. The intertrial interval for all experiments was 500 ms. The participants’ task was to press a button after the ball’s reappearance (detection, experiment 1) or to indicate via button press when the ball would have reached the red bar (extrapolation, experiment 2).

### Data Analyses

We analysed data with repeated measures analyses of variance (ANOVAs) using SPSS (Version 23.0, IBM Corp., United States). For experiment 1 RTs and for experiment 2 absolute deviations from actual arriving time were analysed. For all experiments trials were excluded when no appropriate button was pressed, when flip counts for visual stimulation were not on time (less than 0.15%) or when RT data were outside ± 2 SD on session, participant, and condition level. In total 4.96 and 4.61% of trials were rejected in experiment 1 and 2, respectively. Two-sided *post hoc t*-tests were Bonferroni-corrected and Greenhouse-Geisser correction was used when required.

## Results

### Detection (Experiment 1)

**FIGURE 3:**
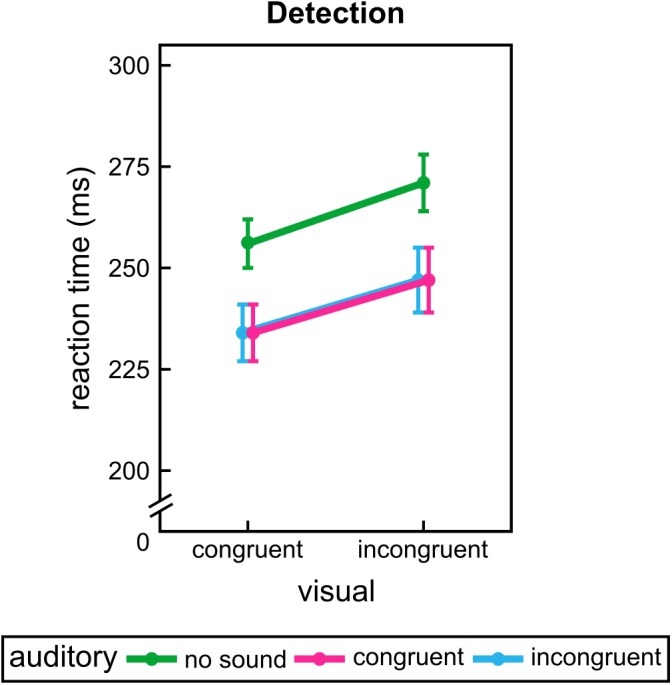
Group mean results of experiment 1. Reaction times for each visual path and sound conditions during detection. Error bars show the standard error of the mean. Points are offset horizontally so that all error bars are visible.

For experiment 1 the results of the repeated measures ANOVA indicated that participants reacted faster for visual congruent paths, *F*(1,35) = 105.00, *p* < 0.001, and were further speeded by additional sounds, *F*(1.25,43.75) = 79.40, *p* < 0.001. Both congruent and incongruent sounds differed from the unimodal condition, *p* < 0.001. However, no difference between auditory conditions was observed, *p* = 1.00. Furthermore, visual information interacted significantly with dimensional presentation, *F* = (1,35), 4.91, *p* = 0.033. *Post hoc* analysis revealed a more pronounced visual facilitation effect (RT difference between visual congruent and incongruent conditions) for pseudo-3D stimulation, *T*(35) = -2.13, *p* = 0.040. All other effects and interactions were not significant, *F* ≤ 2.16, *p* ≥ 0.123. Results of experiment 1 are shown in **Figure 3**.

### Extrapolation (Experiment 2)

**FIGURE 4:**
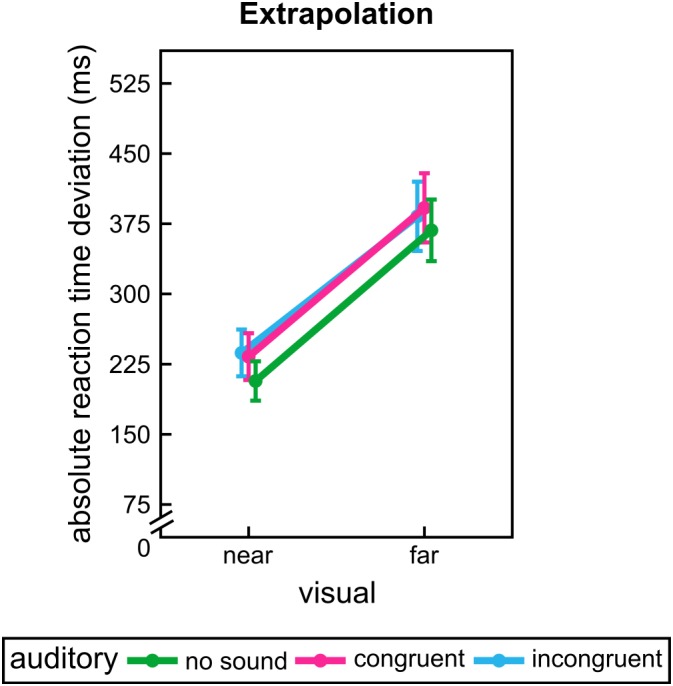
Group mean results of experiment 2. Absolute reaction time deviations for each visual and sound conditions during extrapolation. Error bars show the standard error of the mean. Points are offset horizontally so that all error bars are visible.

For extrapolation again we found a significant influence of the visual factor, *F*(1,31) = 14.76, *p* = 0.001. Participants had a smaller deviation for near extrapolation distance. However, participants seem to be hindered by sounds in this task, *F*(1.06,32.83) = 5.82, *p* = 0.020 (see **Figure 4**). Both auditory conditions led to higher RT deviations than unimodal presentation, *p* = 0.048 (unimodal vs. auditory congruent) vs. *p* = 0.085 (unimodal vs. auditory incongruent). All other effects and interactions were not significant, *F* ≤ 1.06, *p* ≥ 0.330.

It was indeed remarkable that auditory information seems to hinder motion prediction during extrapolation in clear contrast to the beneficial effect during detection. This unexpected result prompted us to explore the data in greater detail. It appeared that some participants benefitted from sounds only at near extrapolation distance whereas others could use them at far distance. To characterise potential interindividual differences between participants in their extrapolation performance for the two distances we chose a cross-validation approach (see e.g., Albrecht et al., 2010; Albrecht and Mattler, 2016; Hagmann and Russo, 2016, for similar approaches): We split our data into two halves and used blocks with even numbers for categorising them into near and far sound users (and vice versa, see below). We classified participants by their auditory facilitation effects (auditory congruent conditions compared to unimodal conditions) for the two extrapolation distances. Consequently, 4 different user groups were possible: 1) general sound users (benefitting from sounds at both distances), 2) near sound users, 3) far sound users and 4) no sound users. Remaining odd blocks were used to analyse RTs for all experimental factors for near and far sound users separately. To cross-validate our approach we repeated this procedure using odd blocks for categorising and even ones for analysing. **Table 1** shows categorising results for user groups based on odd and even block separation. Since the number of participants in the general sound user and no sound user groups were always below 10 we only analysed further the near and far sound user groups.

**Table 1:** Number of participants for each sound user group based on even or odd blocks for group selection.

User group	Even blocks	Odd blocks
General sound users	5	4
Near sound users	11	9
Far sound users	10	11
No sound users	6	8

**FIGURE 5:**
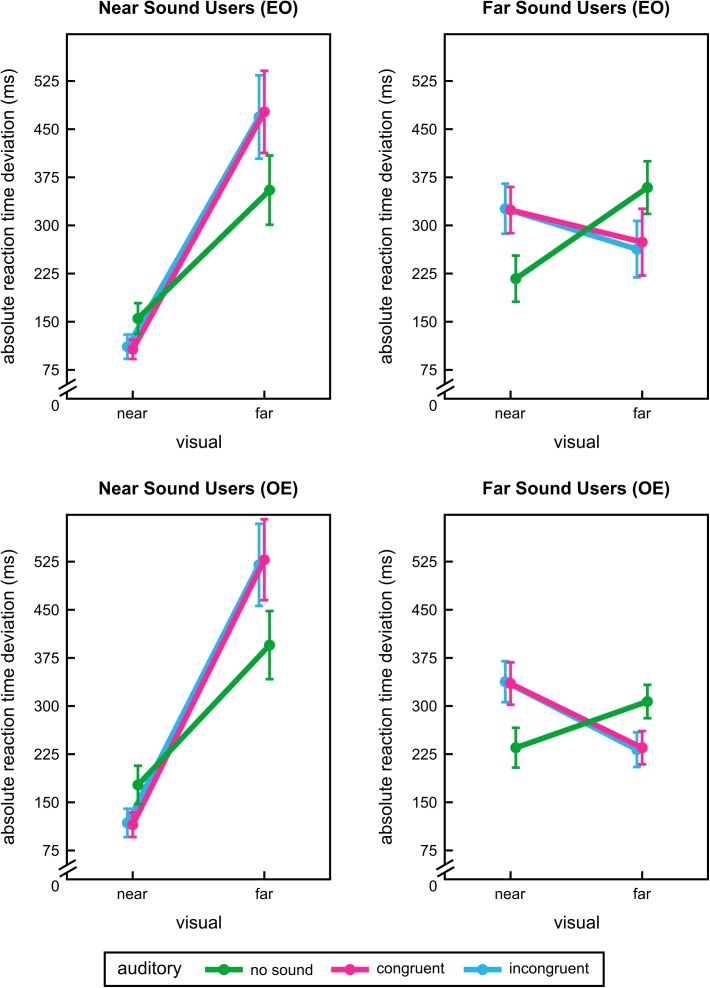
User group results of the exploratory analysis of interindividual differences in experiment 2. Absolute reaction time deviations for each visual and sound conditions during extrapolation for different user groups. Results for near sound users (left panel) and far sound users (right panel) are shown. Near sound users profited from additional sound information at near extrapolation distances, far sound users at far distances. For details of user group segregation see main text. Analyses were conducted twice using a split-half technique: even blocks were used for categorising participants and odd block for analysing data (EO, upper panel) or odd blocks for categorising and even ones for analysing (OE, lower panel). Error bars show the standard error of the mean. Points are offset horizontally so that all error bars are visible.

Analysing the data from odd blocks after subject categorisation based on even blocks (*n* = 11/10 near/far sound users) revealed a significant interaction between visual and auditory stimulation for both near and far sound users, *F*(2,20) = 47.49, *p* < 0.001 and *F*(1.20,10.82) = 40.46, *p* < 0.001 (see **Figure 5** upper panel). For near sound users the effects of vision and audition were also significant: *F*(1,10) = 30.71, *p* < 0.001 and *F*(1.13,11.26) = 9.66, *p* = 0.008. Due to the disordinal interaction effect the main effects were not further considered for interpretation. *Post hoc t*-tests revealed that near sound users were significantly better at the near extrapolation distance when sounds were presented concurrently, *T*(10) = 4.07, *p* = 0.012/*T*(10) = 5.33, *p* < 0.001 (unimodal vs. auditory congruent/incongruent). For far distances near sound users performed better without sound, *T*(10) = -6.52, *p* < 0.001/*T*(10) = -5.43, *p* < 0.001 (unimodal vs. auditory congruent/incongruent). For far sound users the pattern of results was reversed: they responded more accurately at the far extrapolation distance with both sounds, *T*(9) = 5.46, *p* < 0.001/*T*(9) = 7.59, *p* < 0.001 (unimodal vs. auditory congruent/incongruent). At the near extrapolation position their performance was enhanced when no sound was presented, *T*(9) = -4.27, *p* = 0.012/*T*(9) = -4.31, *p* = 0.012 (unimodal vs. auditory congruent/incongruent). For near and far sound users congruent and incongruent sounds did not differ, *p* = 1.00. All other effects and interactions for near and far sound users were not significant, *F* ≤ 1.44, *p* ≥ 0.264.

Data analysis based on even blocks (after user categorisation based on odd blocks, *n* = 9/11 near/far sound users) led to a similar pattern of results. Again, significant interactions between visual and auditory stimulation were found for both near and far sound users, *F*(2,16) = 59.79, *p* < 0.001 and *F*(1.19,11.88) = 29.43, *p* < 0.001 (see **Figure 5** lower panel). Interpretation of visual main effect for near sound users, *F*(1,8) = 38.43, *p* < 0.001, was desisted because of a significant disordinal interaction. All other effects and interactions did not reach significance for both user groups, *F* ≤ 4.04, *p* ≥ 0.075. *Post hoc* comparisons once more indicated that near sound users only profited from sounds at near distance, *T*(8) = 4.55, *p* = 0.012/*T*(8) = 5.74, *p* < 0.001 (unimodal vs. auditory congruent/incongruent), but were worse in motion prediction during additional sound stimulation for far extrapolation distance, *T*(8) = -5.08, *p* = 0.006/*T*(8) = -4.51, *p* = 0.012 (unimodal vs. auditory congruent/incongruent). Far sound users again performed better with sounds when predicting motion at the farther distance, *T*(10) = 4.33, *p* = 0.012/*T*(10) = 5.37, *p* < 0.001 (unimodal vs. auditory congruent/incongruent). For the near extrapolation distance sound again increased RT deviations, *T*(10) = -4.38, *p* = 0.006/*T*(10) = -4.30, *p* = 0.012 (unimodal vs. auditory congruent/incongruent). For both near and far sound users there were no differences between auditory congruent and incongruent conditions, *p* = 1.00. For completeness, all statistical results of all experiments are listed online in the supplementary material (see Supplementary Tables S1–S6).

## Discussion

In this study we tested whether additional auditory information would affect temporal motion prediction as a function of visual disparity in two different tasks. We found that during temporal detection participants profited from congruent visual paths as well as concurrent sounds although the congruence of movement direction between visual and auditory motion did not further modulate RTs. Importantly, visual facilitation for congruent paths was more pronounced during pseudo-3D than real-3D stimulation. In a more complex temporal extrapolation task the overall statistical analysis indicated that sounds hinder motion prediction. However, a detailed exploratory analysis provided evidence for robust interindividual differences during extrapolation. In particular, cross-validation procedures revealed that most participants showed a selective sound-induced benefit either for the near or far extrapolation distance.

In both temporal tasks participants’ behaviour was modulated by auditory co-stimulation. In particular, participants benefitted from both congruent and incongruent sounds to a similar extend. This influence of the auditory information on temporal task performance is in line with previous audiovisual temporal studies (Welch et al., 1986; Fendrich and Corballis, 2001; Repp and Penel, 2002; Recanzone, 2003; Guttman et al., 2005) and may be explained by the superior temporal resolution of the auditory modality. This could suggest that sounds may have simply served as a temporal cue, either at the sustained or phasic level. In experiment 1 participants detected the reoccurrence of the ball after occlusion which coincided with the change in auditory volume (recall that auditory motion was attenuated during occlusion period while the ball was invisible to strengthen audiovisual binding). Hence a simple mechanism triggered by a sudden change in auditory stimulation (e.g., Spence and Driver, 1997) could have caused the effect. This would suggest that sounds act on the phasic level. However, during the extrapolation task (experiment 2), the ball, if visible, would have moved some distance after reappearance from the occluder before passing the near or far line. Nevertheless, a non-spatial facilitating effect of sounds on visual extrapolation was still observed when taking interindividual differences into account. This pattern of results suggest that the sound may have heightened participants’ sustained vigilance rather than providing phasic information.

In contrast, sound direction was less relevant for temporal detection and extrapolation. This might be due to the fact that using the congruency of multisensory direction information was not required (see e.g., Spence, 2013, for a recent review on the lack of spatial influences on non-spatial audiovisual tasks). Alternatively, the spatial information provided by the sounds alone could have been insufficient for successful auditory localisation. However, in an auditory-only pilot experiment (with identical stimuli used in experiments 1 and 2) participants perfectly discriminated movement direction (mean: 99.2%), and even performed well when distinguishing between outer and middle auditory trajectory (except one participant, mean: 87.5%). This pattern of results strongly suggest that auditory spatial information was available but was left unused. Dynamic visual capture might be one explanation for our missing audiovisual congruency effects. In audiovisual motion experiments participants tend to perceive an auditory motion in the same direction as a simultaneously presented visual motion event. If auditory motion is presented alone they had no problems stating direction (Soto-Faraco et al., 2002, 2004). The authors explained this phenomenon with a mechanism based on visual dominance: The visual modality captures auditory perception and participants perceive auditory motion in the same direction as visual motion. Visual capture also occurs for movements in space toward the observer (Kitajima and Yamashita, 1999; Kitagawa and Ichihara, 2002; Alink et al., 2008) and is even more pronounced for looming than receding stimuli (Harrison, 2012). Potentially, auditory motion might also have been captured by visual motion in our experiments so that participants perceived the sounds moving in the same direction as the visual stimulation and therefore auditory motion direction did not influence prediction behaviour.

Importantly, our results of the exploratory analysis provide evidence that interindividual differences further modulate motion prediction performance as the findings of our extrapolation experiment revealed. Here, indeed most participants used auditory information but only a minority were able to do so for both extrapolation distances. Most individuals expressed enhanced performance solely for one distance whereas during extrapolation at a second distance performance decreased. This might also explain why previous studies on audiovisual motion prediction did not find any significant advantage for audiovisual compared to visual-only conditions (Schiff and Oldak, 1990; Hofbauer et al., 2004; Zhou et al., 2007; Hassan, 2012; DeLucia et al., 2016; Keshavarz et al., 2017). It might be possible that their results are confounded by interindividual differences so that no clear advantage of additional auditory information could emerge.

Several previous studies had reported significant influences of individual differences on various multisensory phenomena including point of subjective simultaneity (Eg and Behne, 2015), temporal order judgement (Grabot and van Wassenhove, 2017), intersensory facilitation (Hagmann and Russo, 2016), and McGurk effect (Mallick et al., 2015; Ipser et al., 2017). Our findings extend these observations and demonstrate the influence of individual differences on audiovisual motion prediction.

The fact that most participants only utilised auditory information from one extrapolation distance could be explained by an influence of processing duration on the extrapolation process. Near sound users apparently failed to sustain their extrapolation performance whereas far sound users only profit at later stages during the prediction process. One reason for this variance in response patterns might be different rates at which participants can use audiovisual information. In recent experiments of Sun et al. (2017) participants had to react to audiovisual oscillating fish stimuli and they differed in the rate at which they were able to extract information from audiovisual stimuli for this task. Law et al. (1993) also demonstrated interindividual variability for visual time to arrival judgments and proposed that participants differ in their ability to integrate several visual information sources. It is at least conceivable that our participants may differ in their ability either to extract audiovisual information or to integrate them at different points of time during motion prediction process resulting in differential motion prediction efficiency.

Different strategies or learning histories might also have had an influence on motion prediction performance in our task. In past studies only some participants were able to change their strategy to optimise multisensory performance in a temporal binding window task (Mégevand et al., 2013) and often chose a non-optimal strategy in an audiovisual localization task (Wozny et al., 2010). In our experiments participants may have decided to rely on only one extrapolation distance, therefore did not adjusted their predicting process for both distances and thus behaved non-optimally for one extrapolation distance. This strategy could have been further emphasised by our inter-mixed design in which near and far extrapolation trials were presented in randomised order during blocks. Future studies using blocks for each distance could reveal whether performance for several distances differ from our results because extrapolating movements for only one distance during a longer time interval might coerce all participants to adjust their behaviour accordingly.

Alternatively, individuals could have imagined different ball movements after occlusion. In accord with this notion, Fulvio et al. (2015) reported that participants differed in their prediction whether the ball would move along a linear or quadratic trajectory after occlusion (both movements were theoretically possible), when a ball had followed a quadratic motion trajectory before. Possibly near and far sound users assumed that the ball accelerated or decelerated on its visual trajectory after occlusion and could therefore only benefit from sounds for the one extrapolation distance that matched their assumption. These differences in predictions about moving trajectories could be due to participants’ varying prior experiences. Future studies could investigate to which extent different learning histories might influence motion prediction. For this participants could be trained to expect different motion trajectories like acceleration or deceleration via learned association with unique ball features (e.g., colour). After the training phase coloured balls would move along the same trajectories before disappearing and subjects would again perform an extrapolation task. This way it could be tested whether previously learned associations would affect prediction performance and whether the pattern of results due to colour-trajectory associations resembles the one observed here for near and far users.

While differences in information extraction/integration, the ability to flexibly adjust these processes as well as general experience can be used to describe interindividual differences in motion prediction several underlying mechanisms governing these effects have been proposed. Among them are differences in eye movement patterns (Gurler et al., 2015), task relevant skills like lip reading in McGurk (Strand et al., 2014) and spatial skills/experiences (Schiff and Oldak, 1990). Future research is needed to disentangle between these alternative underlying mechanisms.

Importantly, we found a significant interaction for visual congruence with stimulation dimension in experiment 1 (detection): RTs were shorter for visually congruent stimulation in pseudo-3D sessions than in real-3D sessions. This is in obvious contrast to some previous research: e.g., González et al. (2010) suggested that disparity is an effective cue for motion perception in depth and Harrison et al. (2015) even observed that facilitation effects appear to be stronger in real-3D environments than in experimental set-ups without 3D stimulation. However, in comparison to Harrison et al. (2015) we did not use a discrimination task for auditory motion direction and did not focus on accuracies but rather a detection task with RTs. Possibly, a more pronounced congruence effect in a 3D environment depends on a task which requires the in-depth analysis of spatial properties and is only reflected in answer quality but not in reaction speed during temporal detection.

As an alternative explanation, a deteriorating effect of 3D environments on performance should also be considered. Some studies found a higher sensitivity for 2D motion than 3D motion (Katz et al., 2015; Cooper et al., 2016). In return participants changed their criterion for 3D motion toward more relevant movements toward their heads (Cooper et al., 2016). As a reason for this advantage of 2D over 3D motion Katz et al. (2015) proposed a different temporal integration of motion signals for 2D and 3D movements and a reduced signal-to-noise-ratio during 3D motion sensation. In a manual motion tracking task Bonnen et al. (2017) also demonstrated a lower sensitivity for 3D than 2D motion and they stated that one reason might be a slower disparity processing. This is in line with our findings, particularly because our real-3D and pseudo-3D stimuli only differed in disparity.

Dimension did also not influence performance in our extrapolation experiment. This can be due to the fact that spatial effects in a more naturalistic environment are more pronounced if the spatial dimension is critical for successful task completion (Getzmann and Lewald, 2010; Conrad et al., 2013; Harrison et al., 2015). Potential spatial effects could have been further diminished by the fact that the ball and therefore the moving object was not visible anymore when participants’ reactions were collected in this task. Overall, findings on the effects of 2D vs. 3D stimulation are still debated and appear to be governed by many factors such as task domain (spatial vs. temporal), task demands (detection vs. discrimination vs. extrapolation) and task modality (visual vs. auditory).

## Conclusion

We demonstrated that visual congruency effects in audiovisual motion prediction are more pronounced during pseudo-3D stimulation (without disparity) during a temporal detection task. Therefore, more realistic experimental environments could be used in future motion research to disentangle under which circumstances which 3D depth cues lead to different results than simplified non-3D stimuli. Furthermore, during motion extrapolation we observed individual differences in prediction performance which was evidenced by the point of time at which additional auditory information can optimally be used. Future studies need to take into account interindividual differences when investigating multisensory phenomena and characterise them in even greater detail as these differences cannot be considered as random noise that can sufficiently be reduced by adding more participants and more repetitions.

## Ethics Statement

This study was carried out in accordance with the recommendations of the local ethical committee with written informed consent from all subjects. All subjects gave written informed consent in accordance with the Declaration of Helsinki. The protocol was approved by the local ethical committee.

## Author Contributions

SD designed the study, collected the data, analysed the data, and wrote the manuscript. TN designed the study and wrote the manuscript.

## Conflict of Interest Statement

The authors declare that the research was conducted in the absence of any commercial or financial relationships that could be construed as a potential conflict of interest.

## Footnotes

1 http://www.blender.org
