# Supplementary material for: Temporal Audiovisual Motion Prediction in 2D- vs. 3D-Environments
Source: Front Psychol. 2018 Mar 21;9:368. doi: 10.3389/fpsyg.2018.00368 (PMC5871701; doi:10.3389/fpsyg.2018.00368)
Supplement: Supplementary file 1 [file Data_Sheet_1.pdf]

Supplementary Material

Temporal Audiovisual Motion Prediction in 2D- vs. 3D-Environments

Sandra Dittrich*, Tömme Noesselt

*** Correspondence:** Sandra Dittrich: sandra.dittrich@ovgu.de

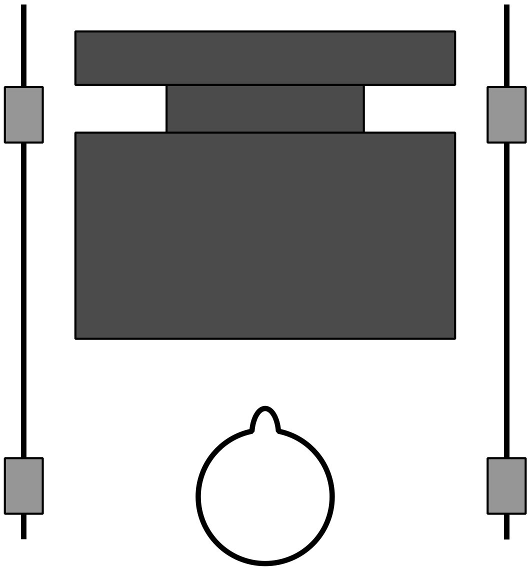

Figure S1. Scheme of experimental set-up (view from above). The participant was placed in front of a stereoscopic monitor consisting of a lower monitor (thin dark grey rectangle) and an upper monitor (thick dark grey rectangle) mounted above the lower one. Four speakers (light grey rectangles) were mounted in head height of the participant (note that vertical speaker position could be adjusted according to each participant's ear position). Speakers were placed on the edges of an imaginary rectangle between the participant's head and the lower monitor. Using this approach virtual sound location could be set to any point between the 4 speaker positions by individually adjusting sound pressure level selectively for each speaker. Additional information about speaker positions and auditory stimulation can be found in the main text.

Statistical results of experiment 1 and 2

Results of reaction time (RT) analyses for experiment 1 (detection) and of absolute RT deviations for experiment 2 (extrapolation). Repeated measures analysis of variance (ANOVA) was conducted with 3 within factors: dimension (pseudo- vs. real-3D), visual (congruent vs. incongruent path after occlusion) and auditory (no sound, congruent vs. incongruent sound movement). For experiment 2 (extrapolation) visual factor was near vs. far extrapolation. Degrees of freedom were Greenhouse-Geisser corrected if necessary and post-hoc comparisons were Bonferroni corrected.

Supplementary Table 1

ANOVA results for experiment 1 (detection).

**effect**	**F**	**df**	**p**
dimension	0.01	1, 35	.917
visual	105.00	1, 35	<.001
auditory	79.40	1.25, 43.75	<.001
dimension x visual	4.91	1, 35	.033
dimension x auditory	1.64	1.46, 51.17	.208
visual x auditory	0.89	2, 70	.414
dimension x visual x auditory	2.16	2, 70	.123

Relevant post-hoc comparisons for interaction effect dimension x visual

Comparison of visually congruent facilitation (difference between visual congruent and visual incongruent conditions): pseudo-3D vs. real-3D: $T(35) = -2.13$, $p = .040$.

Supplementary Table 2

ANOVA results for experiment 2 (extrapolation).

**effect**	**F**	**df**	**p**
dimension	0.03	1, 31	.870
visual	14.76	1, 31	.001
auditory	5.82	1.06, 32.83	.020
dimension x visual	0.21	1, 31	.648
dimension x auditory	1.06	1.30, 40.17	.330
visual x auditory	0.20	1.04, 32.34	.667
dimension x visual x auditory	0.68	1.44, 44.73	.466

Exploratory post-hoc analysis of experiment 2 (extrapolation)

Data were split into two halves (odd and even blocks). One half was used for categorising participants into user groups according to their benefit from sounds for both extrapolation distances (see further details in results section). The other half of the data were used for analysing predicting behaviour. This approach was conducted twice so that even/odd blocks were both used for categorising and analysing respectively.

Supplementary Table 3

ANOVA results for near sound users (even = categorise, odd = analyse).

**effect**	**F**	**df**	**p**
dimension	0.03	1, 10	.873
visual	30.71	1, 10	<.001
auditory	9.66	1.13, 11.26	.008
dimension x visual	0.04	1, 10	.843
dimension x auditory	1.02	2, 20	.380
visual x auditory	47.49	2, 20	<.001
dimension x visual x auditory	0.34	1.21, 12.11	.613

Relevant post-hoc comparisons for interaction effect visual x auditory

Comparisons of near extrapolation conditions: no sound vs. auditory congruent: T(10) = 4.07, p = .012; no sound vs. auditory incongruent: T(10) = 5.33, p < .001; auditory congruent vs. incongruent: T(10) = -0.58, p = 1.0. Comparisons of far extrapolation conditions: no sound vs. auditory congruent: T(10) = -6.52, p < .001, no sound vs. auditory incongruent: T(10) = -5.43, p < .001; auditory congruent vs. incongruent: T(10) = 1.07, p = 1.00.

Supplementary Table 4

ANOVA results for far sound users (even = categorise, odd = analyse).

**effect**	**F**	**df**	**p**
dimension	0.89	1, 9	.370
visual	0.02	1, 9	.897
auditory	0.47	1.19, 10.73	.540
dimension x visual	0.01	1, 9	.953
dimension x auditory	0.19	1.14, 10.25	.705
visual x auditory	40.46	1.20, 10.82	<.001
dimension x visual x auditory	1.44	2, 18	.264

Relevant post-hoc comparisons for interaction effect visual x auditory

Comparisons of near extrapolation conditions: no sound vs. auditory congruent: T(9) = -4.27, p = .012; no sound vs. auditory incongruent: T(9) = -4.31, p = .012; auditory congruent vs. incongruent: T(9) = -0.55, p = 1.0. Comparisons of far extrapolation conditions: no sound vs. auditory congruent: T(9) = 5.46, p < .001, no sound vs. auditory incongruent: T(9) = 7.59, p < .001; auditory congruent vs. incongruent: T(9) = 1.09, p = 1.00.

Supplementary Table 5

ANOVA results for near sound users (odd = categorise, even = analyse).

**effect**	**F**	**df**	**p**
dimension	0.02	1, 8	.888
visual	38.43	1, 8	<.001
auditory	4.04	1.08, 8.64	.075
dimension x visual	0.01	1, 8	.936
dimension x auditory	0.94	2, 16	.412
visual x auditory	59.79	2, 16	<.001
dimension x visual x auditory	1.16	1.19, 9.51	.321

Relevant post-hoc comparisons for interaction effect visual x auditory

Comparisons of near extrapolation conditions: no sound vs. auditory congruent: $T(8) = 4.55$, $p = .012$; no sound vs. auditory incongruent: $T(8) = 5.74$, $p < .001$; auditory congruent vs. incongruent: $T(8) = -0.37$, $p = 1.0$. Comparisons of far extrapolation conditions: no sound vs. auditory congruent: $T(8) = -5.08$, $p = .006$, no sound vs. auditory incongruent: $T(8) = -4.51$, $p = .012$; auditory congruent vs. incongruent: $T(8) = 0.85$, $p = 1.00$.

Supplementary Table 6

ANOVA results for far sound users (odd = categorise, even = analyse).

**effect**	**F**	**df**	**p**
dimension	0.56	1, 10	.472
visual	0.91	1, 10	.363
auditory	1.24	1.26, 12.60	.299
dimension x visual	0.30	1, 10	.598
dimension x auditory	0.73	1.34, 13.42	.448
visual x auditory	29.43	1.19, 11.88	<.001
dimension x visual x auditory	0.18	1.34, 13.43	.750

Relevant post-hoc comparisons for interaction effect visual x auditory

Comparisons of near extrapolation conditions: no sound vs. auditory congruent: $T(10) = -4.38$, $p = .006$; no sound vs. auditory incongruent: $T(10) = -4.30$, $p = .012$; auditory congruent vs. incongruent: $T(10) = -0.49$, $p = 1.0$. Comparisons of far extrapolation conditions: no sound vs. auditory congruent: $T(10) = 4.33$, $p = .012$, no sound vs. auditory incongruent: $T(10) = 5.37$, $p < .001$; auditory congruent vs. incongruent: $T(10) = 0.35$, $p = 1.00$.
